# Epilepsy as a Component of the Dysmorphic–Neurodevelopmental Phenotype in Pediatric Patients with Recurrent Copy Number Variants

**DOI:** 10.3390/genes17030256

**Published:** 2026-02-25

**Authors:** Marlena Młynek, Dorota Wicher, Agata Cieślikowska, Katarzyna Urbańska, Kamila Przywoźna-Zduńczyk, Urszula Zawadzka-Więch, Klaudia Markowska-Krawczyk, Aneta Bal, Sylwia Purwin, Danuta Sielska-Rotblum, Paulina Halat-Wolska, Piotr Iwanowski, Katarzyna Iwanicka-Pronicka, Maria Jędrzejowska, Monika Kowalczyk-Rusak, Justyna Pietrasik, Krystyna Chrzanowska, Dorota Domańska-Pakieła, Katarzyna Kotulska-Jóźwiak, Małgorzata Krajewska-Walasek, Agnieszka Madej-Pilarczyk

**Affiliations:** 1 Department of Medical Genetics, The Children’s Memorial Health Institute, Al. Dzieci Polskich 20, 04-730 Warsaw, Poland; m.mlynek@ipczd.pl (M.M.); d.wicher@ipczd.pl (D.W.); a.cieslikowska@ipczd.pl (A.C.); k.urbanska@ipczd.pl (K.U.); k.zdunczyk@ipczd.pl (K.P.-Z.); u.zawadzka@ipczd.pl (U.Z.-W.); k.markowska@ipczd.pl (K.M.-K.); a.bal@ipczd.pl (A.B.); s.purwin@ipczd.pl (S.P.); d.sielska-rotblum@ipczd.pl (D.S.-R.); p.halat@ipczd.pl (P.H.-W.); p.iwanowski@ipczd.pl (P.I.); k.iwanicka-pronicka@ipczd.pl (K.I.-P.); m.jedrzejowska@ipczd.pl (M.J.); monika.kowalczyk@ipczd.pl (M.K.-R.); j.pietrasik@ipczd.pl (J.P.); k.chrzanowska@ipczd.pl (K.C.); malgorzata.krajewskawalasek@gmail.com (M.K.-W.); 2 Department of Medical Genetics, Institute of Mother and Child, ul. Kasprzaka 17a, 01-211 Warsaw, Poland; 3 Department of Neurology and Epileptology, The Children’s Memorial Health Institute, Al. Dzieci Polskich 20, 04-730 Warsaw, Poland; d.domanska-pakiela@ipczd.pl (D.D.-P.); k.kotulska@ipczd.pl (K.K.-J.)

**Keywords:** array CGH, copy number variants, epilepsy, global developmental delay

## Abstract

**Objective:** Copy number variants (CNVs) overlapping genes associated with neurodevelopmental disorders in patients with epilepsy are particularly concentrated in epilepsy hotspot loci. The aim of this study was to evaluate epilepsy as a component of the dysmorphic–neurodevelopmental phenotype in patients with recurrent CNVs. **Methods:** The study included genetic and clinical data from 177 pediatric patients carrying 17 recurrent CNVs showing well-documented enrichment in epilepsy or associated with genetic OMIM syndromes. **Results:** Epilepsy was diagnosed in 50 of 177 children (28.2%), developmental delay in 147 (83.0%), dysmorphic features in 104 (58.8%), behavioral problems in 62 (35.0%), and congenital anomalies in 55 (31.1%). Among recurrent CNV hotspots, the del16p11.2 BP4–BP5 deletion was the most frequent, occurring in 39 of 177 patients. Ten children (25.6%) with del16p11.2 presented with epilepsy as part of the phenotype. Other frequently represented CNVs included del15q11.2 BP1–BP2 (OMIM #615656; 19/177 patients, 4/19 with epilepsy), del1q21.1 (OMIM #612474; 15/177, 6/15 with epilepsy), del15q13.3 (OMIM #612001; 13/177, 4/13 with epilepsy), and dup16p11.2 (OMIM #614671; 12/177, 1/12 with epilepsy). The highest proportion of epilepsy as a phenotypic component was observed in patients with del1p36 (OMIM #607872; 6/9 patients) and del1q21.1 (OMIM #612474; 6/15 patients). **Conclusions:** Our data support the clinical utility of CNV testing in patients with epilepsy accompanied by additional neurodevelopmental or dysmorphic features, in line with current diagnostic guidelines. The epilepsy-plus phenotype may help clinicians identify patients who are most likely to benefit from CNV analysis.

## 1. Introduction

The term “epilepsy plus” is defined as the occurrence of epilepsy accompanied by comorbid features, including intellectual disability (ID), psychiatric, neurological, and non-neurological manifestations, particularly dysmorphism, which has been proposed in the literature. A recent study of individuals with neurodevelopmental disorders (NDDs) and epilepsy demonstrated comparable frequencies of rare variants among those diagnosed with epileptic encephalopathy and those with NDDs and unspecified epilepsy, supporting the notion that, at the genetic level, epilepsy represents part of the broader spectrum of NDD-associated epilepsies [1].

Previous systematic reviews of clinical series performed in research cohorts of children selected based on an epilepsy diagnosis demonstrated that the burden of deletions and duplications overlapping genes involved in neurodevelopmental processes was particularly concentrated within epilepsy hotspot loci [2,3,4,5,6,7,8,9]. Montanucci et al. analyzed 741,075 individuals and identified 25 copy number variants (CNVs) significantly associated with seizure disorders, linking them to neurodevelopmental and neuropsychiatric phenotypes [10]. This genetic burden is reflected in clinical practice: the diagnostic yield of CNV testing ranges from 5 to 15% in patients with unexplained epilepsy and increases to 15–20% in individuals presenting with unexplained intellectual disability, global developmental delay, autism spectrum disorder (ASD), or multiple congenital anomalies [11].

The literature distinguishes two main types of epilepsy-related CNV: recurrent CNVs with well-documented enrichment in epilepsy and CNVs associated with genetic OMIM syndrome featuring neurological manifestations in which epilepsy may occur, such as Smith–Magenis, Miller–Dieker and Potocki–Lupski syndromes [1]. Cross-cohort analyses indicate that CNV burden varies by epilepsy subtype, contributing to idiopathic generalized, focal, and developmental/epileptic encephalopathies [12,13].

In this study, we aimed to evaluate the contribution of epilepsy to the phenotype of patients with clinically relevant CNVs detected using molecular cytogenetic methods in a hospital-based cohort of children with NDDs.

## 2. Materials and Methods

### 2.1. Data Collection

The retrospective study design was based on patient data were retrieved from the Department of Medical Genetics database at the Children’s Memorial Health Institute (CMHI). The database includes individuals with features of Neurodevelopmental disorders (NDDs), who were referred for cytogenetic and molecular investigation using array Comparative Genomic Hybridization (array CGH), Multiplex Ligation-dependent Probe Amplification (MLPA), and Fluorescence In Situ Hybridization (FISH).

Patients carrying one of 17 known recurrent CNV syndromes with a confirmed association with epilepsy-plus were included in the study (del1p36, del1q21.1, dup1q21.1, del1q44, del3q29, del15q11.2 BP1–BP2, dup15q11.2–q13, del15q13.3, del16p11.2 BP4–BP5, dup16p11.2, del16p13.11, del17p11.2, dup17p11.2, del17p13.3, dup17q12, del22q11.21 distal, dup22q11.21). These CNVs were selected based on robust evidence from the literature demonstrating their recurrent occurrence and well-established association with epilepsy accompanied by additional neurodevelopmental or dysmorphic features, supported by both clinically characterized cohorts and large-scale association analyses, ensuring a well- defined set of CNVs for phenotype correlation in a clinical diagnostic setting. The analysis was restricted to the period between 2013 and 2024, corresponding to the time when array CGH became a routine diagnostic tool in our institution.

Clinical data were collected from their most up-to-date medical records to capture symptoms that may change over time or have late onset and to reflect the most current diagnosis of each patient. General clinical assessments were performed by genetic counsellors or medical specialists.

### 2.2. Genetic Analysis

After obtaining the informed consent, molecular tests were performed using genomic DNA automatically extracted from the peripheral blood leukocytes with either the MagNA Pure LC 2.0 (Roche Diagnostics, Risch-Rotkreuz, Switzerland) or the MagCore Nucleic Acid Extractor HF16Plus (RBC Bioscience, New Taipei City, Taiwan), according to the manufacturer’s protocol. DNA concentration and purity were assessed using Nanodrop 2000 spectrophotometer (Thermo Fisher Scientific, Wilmington, DE, USA).

The whole genome array CGH procedure was performed following the manufacturer’s instructions using Oligo/SNP array CGH (Agilent Technologies, Santa Clara, CA, USA). Both 8 × 60 K CGH and 4 × 180 K CGH+SNP arrays were used. Arrays were scanned using a NimbleGen 200 Microarray Scanner (Roche Nimblegen, Madison, WI, USA). Feature extraction and data analysis were carried out with Agilent CytoGenomics 5.2.1.4 software (Agilent Technologies, Santa Clara, CA, USA) using default analysis settings. Genomic coordinates were interpreted based on the UCSC hg19 assembly.

FISH was performed on fixed metaphase chromosome preparations using locus-specific probes (CytoCell, Cambridge, UK), following the manufacturer’s instructions. Slides were analyzed using a Zeiss Axioscop2 fluorescence microscope, and images were captured and evaluated with Cytovision Karyotyping software version 7.4 (Leica Biosystems, Buffalo Grove, IL, USA).

MLPA analysis was performed using SALSA MLPA KITs P297, P064, P245, ME029, P036+P070 (MRC Holland, Amsterdam, The Netherlands). Denaturation, probe hybridization, ligation and amplification were carried out according to the manufacturer’s recommendations. DNA samples with known copy number alterations were included as a control in each run. Capillary electrophoresis was performed on ABI 3130 and 3500 genetic analyzers, and data were initially processed using Gene Mapper software v3.7 and v4.1 (Applied Biosystems, Foster City, CA, USA). Further analysis was conducted using GeneMarker version 2.2.0 (Soft Genetics, LLC, State College, PA, USA) and Coffalyser.Net [v.240129.0000] (MRC Holland, Amsterdam, The Netherlands). Peak ratio thresholds ranging from 0.7 to 1.3 were applied for the detection of copy number losses and gains.

Parental testing was performed whenever available for patients with suspected pathogenic CNVs, following informed consent. MLPA was used to confirm CNVs and determine their mode of CNV inheritance. In cases involving terminal chromosomal fragments, parental FISH analysis was performed to exclude balanced chromosomal abnormalities.

The pathogenicity of CNVs was predicted based on their size and gene content, in accordance with the American College of Medical Genetics and Genomics (ACMG) guidelines for interpretation of postnatal CNVs [14].

## 3. Results

We selected 177 patients carrying one of 17 known CNV syndromes with confirmed association with epilepsy from a cohort of children with NDDs (Table 1). The clinical phenotypes and the inheritance of the CNV (when investigated) are summarized in Table S1 in Supplementary Materials.

Epilepsy was diagnosed in 50 of 177 children (28.2%) (Table 2). The epilepsy phenotypes were heterogeneous, with the most common types including focal epilepsy (both drug-resistant and with preserved awareness), West syndrome (infantile spasms), generalized epilepsy with absence-like episodes, and developmental and epileptic encephalopathy. Among the 50 patients with epilepsy, all presented with developmental impairment; 21 (42%) had behavioral problems, and 35 (70%) exhibited dysmorphic features. Overall, developmental delay was diagnosed in 147 (83.0%) children, dysmorphic features in 104 (58.8%), behavioral problems in 62 (35.0%), and congenital anomalies in 55 (31.1%).

Among recurrent CNV hotspots, the 16p11.2 BP4–BP5 deletion was the most frequent, occurring in 39 of 177 patients. Epilepsy was present in 10 children (25.6%) with the 16p11.2 deletion. Other frequently observed CNVs included the 15q11.2 BP1–BP2 deletion (OMIM #615656; 19/177 patients, 4/19 with epilepsy), the 1q21.1 deletion (OMIM #612474; 15/177, 6/15 with epilepsy), the 15q13.3 deletion (OMIM #612001; 13/177 patients, 4/13 with epilepsy), and the 16p11.2 duplication (OMIM #614671; 12/177 patients, 1/12 with epilepsy).

The highest proportion of epilepsy as a phenotypic component was observed in patients with 1p36 deletion syndrome (OMIM #607872; 6/9 patients). In this subgroup, all patients carried pure terminal deletions, with deletion sizes ranging from 1.6 Mb to 10.9 Mb. In one case, the precise size could not be determined, and the diagnosis was established by FISH analysis. In all cases in which parental analysis was available, the deletion occurred de novo. A similarly high proportion of epilepsy was observed in patients with 1q21.1 deletion syndrome (OMIM #612474; 6/15 patients).

Epilepsy was identified in 1/12 patients with dup16p11.2, 1/4 with del16p13.11, 1/5 patients with dup17q12, and 1/3 with del22q11.21. In contrast, none of the patients with 22q11.2 duplication (OMIM #608363; *n* = 13) or 3q29 deletion (OMIM #609425; *n* = 8) had epilepsy. Three patients (1.7%) carried two pathogenic or likely pathogenic CNVs.

We identified three genomic loci in which CNVs were consistently associated with epilepsy as a component of the phenotypic spectrum in all affected patients: 1q44 deletion (OMIM #612337; 5/5 patients), 17p11.2 duplication (Potocki–Lupski syndrome, OMIM #610883; 2/2 patients), and 17p13.3 deletion (Miller–Dieker lissencephaly deletion syndrome, OMIM #247200; 3/3 patients). In patients with del1q44, minimum and maximum deletion sizes varied from 1.1 Mb to 11. Mb. Three deletions were terminal and two interstitial.

## 4. Discussion

The influence of genetic variation on the etiology of epilepsy, particularly childhood-onset epilepsy, has been extensively studied over many years. Over the past decade, numerous genetic studies have established that single nucleotide variants can confer susceptibility to epilepsy or directly cause the disorder [16,17]. In contrast, the genetic mechanisms by which CNVs contribute to epilepsy or other developmental disorders remain incompletely understood. In the case of microdeletions, several mechanisms have been proposed to explain their incomplete penetrance, including the unmasking of a recessive allele, the presence of a non-coding regulatory variant within the deleted region, or the coexistence of a second large CNV that may contribute to a more severe phenotype [18].

Our findings support the role of large recurrent CNVs as important genetic risk factors for epilepsy and highlight several variants encompassing established epilepsy-associated genes. Numerous recurrent CNVs have been linked to a broad spectrum of neurodevelopmental phenotypes, with epilepsy representing a frequent and clinically relevant manifestation.

The most frequent CNV in our cohort was the 16p11.2 deletion, accounting for 21.8% of recurrent CNVs, consistent with previous meta-analytic data [1]. Seizures and epilepsy are well-recognized features of 16p11.2 CNVs, occurring in approximately 24% and 18% of deletion carriers, respectively, with heterogeneous seizure types. In contrast, duplication carriers demonstrate a lower risk (approximately 15% for seizures and 10% for epilepsy) [19]. In our cohort, epilepsy was observed in 10 of 39 individuals with the 16p11.2 deletion and 1 of 12 individuals with the duplication, reflecting this reported variability.

Large-scale neuroimaging studies of distal 1q21.1 CNVs showed structural brain alterations and associated cognitive deficits [20]. In our patients, NDDs were observed in 12 of 15 patients (80%) carrying the 1q21.1 deletion, microcephaly was present in 6 of 15 patients (40%), and Chiari malformation was also identified. Epilepsy was diagnosed in 40% of individuals with the 1q21.1 deletion, a proportion slightly higher than estimates reported in patient registries and GeneReviews (18–35%) [21,22]. Seizures appear to be less frequent in 1q21.1 duplication carriers, with epilepsy observed in 1 of 10 patients (10%) in our cohort, consistent with previous reports [23].

Recurrent CNVs at 16p13.11 and 22q11.2 have previously been associated with both generalized and focal epilepsy in large cohort studies [3,12,24,25]. In our cohort, epilepsy was observed in 1 of 4 patients with del16p13.11 and in 1 of 13 patients with dup22q11.21. The type of epilepsy varied across affected individuals, underscoring that seizure phenotypes in individual patients may differ from cohort-based expectations and reflect a broad spectrum of neurological outcomes.

In the case of dup22q11.2, similarly to dup 15q11.2q13, dup17q12 and dup16p11.2, the frequent co-occurrence of autism spectrum disorder (ASD) and behavioral problems within the neurodevelopmental phenotype is noteworthy. Such associations have previously been reported in 18–30% of children with epilepsy who meet diagnostic criteria for ASD [26,27]. In our cohort, the phenotypic expression across these CNVs was highly variable. Although most carriers exhibited ASD-related behavioral and developmental features, epilepsy was diagnosed in only a subset of patients: 2 of 10 cases with dup15q11.2q13, 1 of 4 with del16p13.11, 1 of 5 with dup17q12, and 1 of 12 with dup16p11.2. These findings highlight the frequent yet CNV-specific and variable co-occurrence of ASD and epilepsy.

Among 19 patients with 15q11.2 deletion, epilepsy was diagnosed in 4 of them (21%), consistent with previous clinical series [28]. None of the carrier relatives reported seizures. Large cohort studies suggest that 15q11.2 deletions should be interpreted cautiously in the context of epilepsy risk [12]. The BP1–BP2 region contains four evolutionarily conserved, non-imprinted genes expressed in the central nervous system, among which *CYFIP1* is considered a primary candidate gene underlying the observed neurodevelopmental phenotypes. Although this CNV is characterized by reduced penetrance (below 10%) and a relatively high frequency in the general population (>0.1%), substantial evidence supports its association with neurodevelopmental disorders and reduced cognitive abilities, rendering its clinical classification a subject of ongoing debate [28,29].

Similarly, for the del15q13.3 recurrent deletion, the frequency of epilepsy in our cohort corresponds to that reported in the literature. Individuals with this deletion may present a broad range of clinical manifestations, with epilepsy developing in approximately 30% of cases [30]. Additional symptoms due to unmasking of a recessive allele of the *TRPM1* due to del15q13.3 deletion have been described [31].

Among the syndromic pathogenic autosomal CNVs identified in our cohort, epilepsy was observed across all three contiguous gene syndromes analyzed, although with varying frequencies. Seizures occurred in 3 of 9 patients with Smith–Magenis syndrome, consistent with previously reported prevalence estimates and supporting epilepsy as a variable component of the neurological phenotype associated with this condition [32]. All three individuals with Miller–Dieker syndrome developed epilepsy, in line with the well-established association between lissencephaly and early-onset seizures. In Potocki–Lupski syndrome, epilepsy was observed in both affected individuals; however, seizures have been reported as a relatively infrequent manifestation of this syndrome, occurring in approximately 5–10% of patients. Given the limited sample size, this observation should be interpreted with caution and may reflect ascertainment bias rather than an increased baseline seizure risk associated with dup17p11.2 [33].

A detailed phenotypic comparison of patients with distinct 1q44 deletions in our cohort further illustrates gene-specific contributions within this region. Notably, all patients with 1q44 microdeletion presented with epilepsy. The patient harboring a de novo interstitial deletion involving *ZBTB18* and *AKT3*, but sparing *HNRNPU*, exhibited global developmental delay, speech delay, microcephaly, dysmorphic features, and seizures. In contrast, a patient with a deletion restricted to *HNRNPU* demonstrated severe, drug-resistant epilepsy, extensive micro-/pachygyria, profound intellectual disability, and marked psychomotor delay. These findings are consistent with previous reports [15] implicating *AKT3* and *ZBTB18* primarily in brain growth and craniofacial development, while highlighting *HNRNPU* as a critical contributor to cortical malformations and severe epileptic encephalopathy within the 1q44 microdeletion spectrum.

The 1p36 terminal deletion syndrome represents a significant cause of epilepsy. Our findings confirm that more than half of affected children develop epilepsy and that deletion size is not necessarily correlated with phenotypic severity [34]. Both distal and proximal 1p36 critical regions have been described [35], encompassing genes whose haploinsufficiency contributes to the neurodevelopmental phenotype. All patients in our cohort shared deletions involving the distal 1p36 critical region, encompassing *GABRD* and *GNB1* genes. In two patients, the deletion extended proximally to include *KCNAB2*; epilepsy was observed in one of these individuals.

Interestingly, none of the patients with recurrent CNVs involving del3q29 (*n* = 8) or dup22q11.21 (*n* = 13) in our cohort presented with epilepsy, despite previous reports indicating that seizures may occur in a subset of individuals with these syndromes [36,37]. This observation underscores the variable penetrance of epileptic manifestations in recurrent CNVs and highlights the potential role of additional genetic, epigenetic, or environmental modifiers of seizure risk. Importantly, the absence of epilepsy did not preclude the presence of significant neurodevelopmental or behavioral phenotypes in these patients.

Three individuals in our cohort carried two pathogenic CNV hotspots: one with dup1q21.1 in combination with del15q13.3, one with dup15q11.2q13 together with dup16p11.2, and one with dup1q21.1 together with del16p11.2. None of these patients developed epilepsy, despite the presence of multiple potentially pathogenic variants. This finding suggests that seizure risk is not simply additive in the context of multiple CNVs and highlights the complex interplay of genetic and possibly environmental factors underlying epileptogenesis. Nevertheless, all three patients exhibited significant neurodevelopmental and behavioral abnormalities, reinforcing the pleiotropic effects of CNVs beyond seizure susceptibility.

Our study has several limitations. The study was performed in a hospital-based cohort of children with neurodevelopmental disorders who were referred for molecular testing based on clinical genetic evaluation, which may have introduced selection bias. Additionally, the retrospective nature of data collection may limit the completeness and consistency of clinical information. The study population was relatively homogeneous, and, therefore, the findings may not be generalizable to populations of different ethnic or geographic backgrounds. The use of different genetic testing methodologies over time may have influenced detection rates and the spectrum of identified CNV. Furthermore, some patients were evaluated using FISH or MLPA alone, limiting the ability to detect additional potentially contributory CVNs. Retrospective data may also underestimate true population prevalence due to incomplete documentation, asymptomatic or late-onset presentations, and variability in healthcare access. This study focused exclusively on CNVs with previously established associations with epilepsy; therefore, the observed frequencies reflect this pre-selection and should not be interpreted as evidence for novel associations or as estimates of population-level risk. The primary contribution of our findings is the confirmation of known associations in a clinical cohort and the provision of descriptive phenotypic data.

Lastly, the high degree of pleiotropy observed among seizure-associated CNVs suggests that these variants disrupt neurodevelopmental processes in a relatively nonspecific manner, thereby contributing to a wide spectrum of neurodevelopmental disorders. Recent studies have demonstrated interactions between CNVs and the polygenic background, showing that large deletions often affect additional loss-of-function-intolerant genes beyond the primary disease-associated gene [38]. Continued advances in whole-genome sequencing technologies will enable more comprehensive assessment of the contribution of a broader spectrum of rare genetic variants, including balanced rearrangements, small CNVs, and short tandem repeats, which may act as additional or modifying factors in epilepsy.

Overall, the mechanisms by which large, polygenic microdeletions increase risk for neurodevelopmental disorders remain incompletely understood. The marked phenotypic variability associated with recurrent CNVs poses significant challenges for genetic counseling, as individuals inheriting the same CNV may present with milder, more severe, or no clinical manifestations. At present, no reliable biomarkers or predictive tests are available to accurately forecast these outcomes.

## 5. Conclusions

Our study confirms the clinical utility of CNV testing in patients with epilepsy accompanied by additional neurodevelopmental features, supporting current diagnostic guidelines. Recurrent CNVs act as important risk factors characterized by incomplete penetrance and marked phenotypic variability. Our findings indicate that epilepsy with co-occurring neurodevelopmental or dysmorphic features should prompt consideration of CNV analysis as an early step in the diagnostic algorithm.

## Figures and Tables

**Table 1:** Overview of analyzed CNVs, associated syndromes, genomic boundaries, and epilepsy-related genes.

Samples [*n*]	CNV Type	Syndrome	OMIM or Reference	Reference Boundaries [GRCh37]	Epilepsy/Brain-Related Disease Gene
9	del 1p36	Chromosome 1p36 deletion syndrome	#607872	1:10 001- 27 926 511	*GNB1*, *KCNAB2*, *GABRD*
15	del 1q21.1	Chromosome 1q21.1 deletion syndrome	#612474	1:146 577 485-147 394 506	*GJA5*, *GJA8*, *CHD1L*
9	**dup 1q21.1**	Chromosome 1q21.1 duplication syndrome	#612475	1:146 577 485-147 394 506	*GJA5*, *GJA8*, *CHD1L*
5	del 1q44	Chromosome 1q44 microdeletion syndrome	[15]	1:243 287 729-245 318 287	*HNRNPU*, *ZBTB18*, *AKT3*
8	del 3q29	Chromosome 3q29 microdeletion syndrome	#609425	3:195 756 053-197 344 662	*DLG1*
19	del 15q11.2 BP1-BP2	Chromosome 15q11.2 deletion syndrome	#615656	15:22 832 518-23 090 897	*CYFIP1*
10	dup 15q11.2-q13	Chromosome 15q11-q13 duplication syndrome	#608636	15:23 747 995-28 379 874	*SNRPN*
13	del 15q13.3	Chromosome 15q13.3 deletion syndrome	#612001	15:31 192 888-32 445 405	*CHRNA7*
39	del 16p11.2 BP4-BP5	Chromosome 16p11.2 deletion syndrome	#611913	16:29 649 996-30 199 852	*PRRT2*
12	dup 16p11.2 BP4-BP5	Chromosome 16p11.2 duplication syndrome	#614671	16:29 649 996-30 199 852	*PRRT2*
4	del 16p13.11	Chromosome 16p13.11 deletion syndrome	[6]	16:15 511 710-16 292 265	*MYH11*
9	del 17p11.2	Smith–Magenis Syndrome	#182290	17:16 810 027-20 213 202	*RAI1*
2	dup 17p11.2	Potocki–Lupski Syndrome	#610883	17:16 810 027-20 213 202	*RAI1*
3	del 17p13.3	Miller–Dieker lissencephaly deletion syndrome	#247200	17:1 247 832- 2 588 909	*PAFAH1B1*, *YWHAE*
5	dup 17q12	Chromosome 17q12 duplication syndrome	#614526	17:34 815 071-36 192 489	*SYNRG*
3	del 22q11.21	Chromosome 22q11.2 deletion syndrome, distal	#611867	22:21 917 116-23 649 111	*TBX1*
13	dup 22q11.21	Chromosome 22q11.2 duplication syndrome	#608363	22:18 912 230-21 465 672	*TBX1*

**Table 2:** Genetic and phenotypic characteristics of children with recurrent CNV hotspots related to clinically diagnosed epilepsy.

Syndrome	Patient	Sex	Neurodevelopmental Disorder	Dysmorphic Features	Epilepsy/Brain Anomalies	Other Anomalies	Inheritance
**del 1p36**	#2	F	Delayed psychomotor development	+	Focal epilepsy, CNS defect	Cardiomyopathy due to left ventricular non-compaction	de novo
	#3	F	Delayed psychomotor development and hypotonia	+	Polymicrogyria and drug-resistant epilepsy		de novo
	#4	M	Delayed psychomotor development, decreased muscle tone, and behavioral problems	+	West Syndrome	Cardiomyopathy due to left ventricular non-compaction	de novo
	#5	M	Delayed psychomotor development and hypotonia	+	Epilepsy	Binocular cataracts and left transverse sulcus	NT
	#6	F	Global developmental delay, ID	+	Epilepsy		de novo
	#9	F	Delayed psychomotor development and abnormal muscle tone	+	Epilepsy, hypoplasia of the corpus callosum, and hydrocephalus	Ebstein’s anomaly	de novo
**del 1q21.1**	#10	F	Delayed psychomotor development	+	Focal epilepsy	Extreme prematurity	Paternal
	#13	F		+	Unspecified epilepsy with seizures and loss of consciousness	Patent ductus arteriosus, obesity, sinus tachycardia, bladder dysfunction, juvenile spondyloarthropathy, lymphedema	Maternal
	#17	M	Hyperactivity	+	Epilepsy	Urinary tract defect, nystagmus, microcephaly, iris coloboma, optic nerve atrophy	de novo
	#18	F	Delayed psychomotor development and behavioral problems	+	Epilepsy	Microcephaly	Maternal
	#20	M	Delayed speech development, ID, and behavioral problems		Focal epilepsy with preserved awareness	Microcephaly	de novo
	#24	F	Delayed speech development, ID, and behavioral problems	+	Epilepsy	Microcephaly	Maternal
**dup 1q21.1**	#28	M	Autism spectrum disorder		Epilepsy		Not Maternal
**del 1q44**	#34	F	Delayed psychomotor development,		Drug-resistant epilepsy of unknown etiology and developmental encephalopathy	Right ear defect, Hearing loss	NT
	#35	F	Delayed motor and speech development	+	Epilepsy, myelination disorder, atrophy of the cerebellar, Agenesis of the corpus callosum	Microcephaly, Clubfoot, Strabismus	NT
	#36	M	Delayed psychomotor development and severe ID	+	Drug-resistant epilepsy of organic etiology—brain defect—extensive micro/pachygyria,		de novo
	#37	F	Behavioral disorders	+	Epilepsy	Heart defect, microcephaly, and microsomia	Not Maternal
	#38	M	Delayed psychomotor development and delayed speech development	+	Epilepsy	Microcephaly	de novo
**del 15q11.2**	#52	M	Delayed psychomotor development, ID		Focal Drug-resistant epilepsy	Hypothyroidism	Not Maternal
	#55	F	Learning difficulties	+	Epilepsy		Maternal
	#60	F	Moderate ID		Epilepsy		NT
	#64	M	Delayed psychomotor development and hypotonia	+	Epilepsy, Dilatation of the ventricular system	Microcephaly	Paternal
**dup 15q11.2-q13**	#68	F	Delayed psychomotor development and autism spectrum disorder	+	Epilepsy	Microcephaly	de novo
	#70	F	Delayed psychomotor development	+	West Syndrome	Cleft palate	de novo
**del 15q13.3**	#78	M	Delayed psychomotor development	+	Seizure-like episodes with unresponsiveness	Joint laxity, * significant myopia, amblyopia, strabismus, and nystagmus	de novo
	#82	F			Epilepsy		NT
	#83	M	Moderate ID, Delayed motor and speech development, Behavioral disorders		Epilepsy		NT
	#84	M	Delayed speech development and delayed psychomotor development		Epilepsy, Frontal lobe abnormalities		NT
**del 16p11.2**	#88	M	Speech development disorders, increased muscle tone, and behavioral disorders	+	Epilepsy with absences		de novo
	#98	M	ID	+	Epilepsy		Maternal
	#100	M	Mild ID		Epilepsy		Maternal
	#101	F	Mild ID, Autism	+	Epilepsy	Prematurity, obesity,* and short stature with disturbed body proportions	NT
	#108	M	Impaired speech development	+	Epilepsy		NT
	#113	F	Normal development		Epilepsy		de novo
	#114	F	Delayed psychomotor and speech development		Epileptic seizures from the age of 4 months		NT
	#119	M	ID		epilepsy	Hyperthyroidism	de novo
	#120	M	Moderate ID and delayed speech development	+	Seizures and abnormal EEG with bilateral diffuse changes	Heart defect	NT
	#122	M	Delayed psychomotor and speech development and behavioral disorders, Mild ID	+	Epilepsy		de novo
**del 16p13.11**	#139	M	Lack of speech development and behavioral disorders		Drug-resistant epilepsy		NT
**dup 16p11.2**	#133	F	Mild ID, Depression, and tetany		Epilepsy		de novo
**del 17p11.2**	#146	M	Moderate ID, delayed psychomotor and speech development, and behavioral disorders	+	Epilepsy	Hypothyroidism	NT
	#147	F	Delayed psychomotor development and moderate ID	+	Epilepsy		NT
	#148	F	Moderate ID and behavioral disorders	+	Epilepsy	Generalized joint laxity	NT
**dup 17p11.2**	#152	F	ID	+	Seizures and abnormal EEG	Hyperopia	NT
	#153	M	Delayed psychomotor development	+	Epilepsy		NT
**del 17p13.3**	#154	F	Severe ID		Epilepsy and pachygyria	Microcephaly	de novo
	#155	M	Delayed psychomotor development and quadriplegia		Drug-resistant epilepsy and lissencephaly		de novo
	#156	M	moderate ID		Epilepsy, Pachygyria		NT
**dup 17q12**	#157	M	Disharmonious psychomotor and speech development		Febrile seizures with loss of consciousness	Cardiac arrhythmia syncope	NT
**del 22q11.2**	#163	M	Moderate ID and autism	+	Epilepsy	Heart defect and obesity	de novo

Abbreviations: ID—Intellectual disability, CNS—Central Nervous System, NT—not tested. * Symptoms correlated with an additional genetic change identified in the patient.

## Data Availability

The original contributions presented in this study are included in the article/Supplementary Materials. Further inquiries can be directed to the corresponding author.

## Supplementary Materials

The following supporting information can be downloaded at https://www.mdpi.com/article/10.3390/genes17030256/s1: Supplementary Table S1. Comprehensive overview of recurrent CNVs and clinical features of the studied cohort.

## Abbreviations

The following abbreviations are used in this manuscript:

ID	Intellectual disability
NDD	Neurodevelopmental delay
CNV	Copy Number Variants
array CGH	Array Comparative Genomic Hybridization
MLPA	Multiplex Ligation-dependent Probe Amplification
FISH	Fluorescence In Situ Hybridization
ACMG	American College of Medical Genetics and Genomics
